# Supplementary material for: Undiagnosed COVID-19 in households with a child with mitochondrial disease
Source: medRxiv. 2022 Mar 23:2022.03.21.22272358. Preprint. [Version 1] doi: 10.1101/2022.03.21.22272358 (PMC8963689; doi:10.1101/2022.03.21.22272358)
Supplement: Supplement 1 [file media-1.docx]

**SUPPLEMENTAL DATA**

**Figure S1: Map of the United States showing number of cases by county.** Seven-day averages per 100K population were compiled for families on the day of collection of samples. Some counties (e.g., Tarrant County, TX) contained more than one family.
